# Use of a Mini-Scleral Lens in Patients with Keratoconus

**DOI:** 10.4274/tjo.galenos.2020.56804

**Published:** 2020-12-29

**Authors:** Semra Akkaya Turhan, Deniz Özarslan Özcan, Ebru Toker

**Affiliations:** 1 Marmara University Faculty of Medicine, Department of Ophthalmology, İstanbul, Turkey; 2 Mustafa Kemal Üniversitesi Tıp Fakültesi, Göz Hastalıkları Anabilim Dalı, Hatay, Türkiye

**Keywords:** Visual performance, keratoconus, mini-scleral lens

## Abstract

**Objectives:**

To assess the visual performance of a mini-scleral lens in patients with keratoconus and to evaluate its fit by optical coherence tomography (OCT).

**Materials and Methods:**

Twenty-nine eyes of 24 patients with keratoconus were fitted with a mini-scleral lens (Esclera; Mediphacos Inc., Belo Horizonte, Brazil). Diagnostic lenses were used in the initial fitting process. The lens fit was evaluated by the fluorescein pattern and also by anterior segment OCT (RTVue, Optovue Inc., Fremont, CA). Within 30-45 minutes after insertion, the lens fit parameters including central corneal and limbal clearance, and peripheral landing zone alignment were evaluated by OCT. High- and low-contrast visual acuity (VA), subjective performance for comfort and vision (5-point Likert scale), and overall satisfaction with the lens (100 mm visual analog scale [VAS]) were measured before and after lens wear.

**Results:**

The mean decimal high-contrast VA (best spectacle-corrected VA: 0.40±0.14 vs VA with the scleral lens: 0.93±0.12, p<0.0001) and low-contrast VA (best spectacle-corrected VA: 0.60±0.24 vs VA with the scleral lens: 1.15±0.18, p<0.0001) significantly improved with lens wear. The mean central corneal clearance was 120.7±24.5 μm. There were no correlations between the keratometric values and the sagittal depth of the scleral lens. The mean number of trial lenses required for ideal fit was 2.2 lenses (range: 1-8). Patients reported high scores for comfort (mean score: 4.69; range: 4-5), vision (mean score: 4.62; range: 3-5) and overall satisfaction with the lens (mean VAS score: 88.1; range: 70-100).

**Conclusion:**

The mini-scleral lens provided good high- and low-contrast visual acuity and high patient satisfaction in patients with keratoconus. Anterior segment OCT imaging facilitated the evaluation of the fit.

## Introduction

Optical correction methods are used to improve the visual function of keratoconus patients. Progression of the disease leads to complex optical aberrations.^1,2^ Rigid contact lenses can be used to reduce these aberrations.^3^ However, despite the optical benefits provided by rigid contact lenses, they may not be a good fit for every patient. Lens decentration due to increased corneal irregularity, corneal scarring, and patient discomfort are important problems in more advanced cases.^4^ Today, scleral lenses are a good option that can be used to prevent or delay surgery, especially when other lens options have been unsuccessful.^5^ The tear reservoir between the scleral contact lens and cornea provides optical neutralization of irregular corneas, corneal hydration in ocular surface diseases, and high optical quality for vision and therapeutic applications.^6,7,8^

The key in scleral lens fitting is to position the lens parallel to the scleral contour, leaving a gap over the cornea and limbus but without creating pressure on the conjunctiva or edge lift. The fitting of scleral lenses differs from other lenses because it is based on sagittal height. An apical clearance between 100 and 400 µm is recommended depending on the material and design of the lens used.^9^ Anterior segment optical coherence tomography (OCT) provides valuable information in the quantitative determination of clearance at each meridian from the central cornea to the limbus.^10^ Evaluating the fit with OCT enables better lens fit and comfort to be achieved with the use of fewer trial lenses.^11^ The aim of this study was to evaluate the visual performance and fit of a mini-scleral lens in keratoconus patients with OCT.

## Materials and Methods

This retrospective study included 29 eyes of 24 keratoconus patients fitted with mini-scleral lenses (Esclera; Mediphacos Ltd., Belo Horizonte, Brazil).

Trial lenses were used for initial fit assessment. Points to consider during scleral lens fitting include:^9^

**Figure 1:**
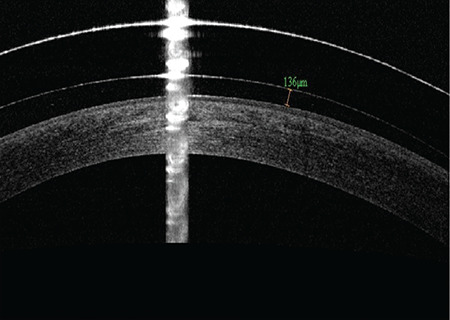
Measurement of apical clearance with optical coherence tomography

**Figure 2:**
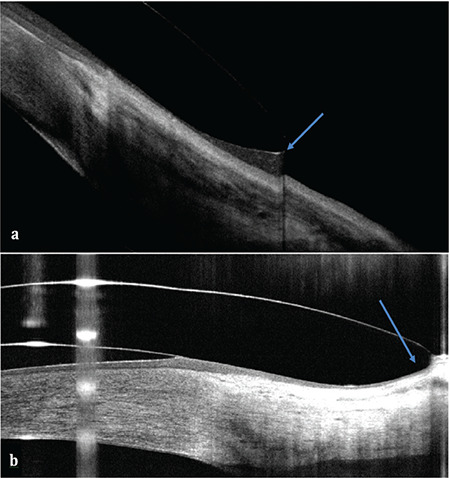
a) Edge lift: the lens edge is raised off the sclera (blue arrow). b) Conjunctival billowing: the lens compresses the conjunctival epithelium, causing it to thin and gather at the lens edge (blue arrow)

1. The scleral lens should extend 2 mm beyond the limbus.

2. The minimum sagittal depth should ensure central clearance. If there is apical contact, sagittal depth should be increased to achieve central clearance of at least 100 µm (Figure 1).

3. The lens edges should be checked to ensure they are not too raised or tight on the sclera (Figure 2a, b).

4. Final refraction should be evaluated through the lens.

**Figure 3:**
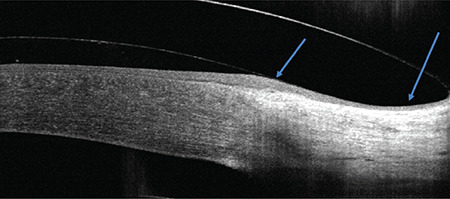
Peripheral edge fit: the lens edge should not have too much lift or be too tight on the sclera (ideal fit)

Lens fit was evaluated by fluorescein pattern and anterior segment OCT (RTVue, Optovue Inc., Fremont, CA) imaging. At 30-45 minutes after lens application, lens fit parameters including central clearance, limbal clearance, and peripheral fit (no conjunctival compression or blanching, no edge lift) were evaluated with OCT. An ideal peripheral fit is shown in Figure 3.

All patients underwent a complete ophthalmologic examination. High-contrast visual acuity (VA) was measured in decimal using a standard Snellen chart at a distance of 6 meters. Low-contrast VA was measured using the Pelli-Robson Test (Vision Chart v 1.3.0 CSO, Florence, Italy) from a distance of 3 meters.^12^ The Pelli-Robson Test, which includes optotypes of varying sizes and contrasts, consists of 16 sets of 3 letters at the same contrast, which decreases by 0.15 logCS between each set. Topographic measurements were made using a Scheimpflug camera system (Pentacam; Oculus Optikgerä te GmbH, Wetzlar, Germany). The flat meridian (K1), steep meridian (K2), and maximum keratometric value (Kmax) were recorded in diopters (D). Keratoconus staging was performed using the Amsler-Krumeich classification system.^13^ High- and low-contrast VA, subjective performance for comfort and vision (5-point Likert scale), and overall satisfaction on a 100-mm visual analog scale (VAS) were evaluated before and after lens wear.

### Statistics Analysis

The study data were evaluated using SPSS version 21.0 (IBM Corp., Armonk, NY) software. The Kolmogorov-Smirnov test was used to evaluate whether the data showed normal distribution. Parameters before and after scleral lens wear were compared using Wilcoxon test, with a p value <0.05 considered statistically significant. The relationship between keratometric values and sagittal depth was evaluated using Spearman correlation test.

## Results

**Figure 4:**
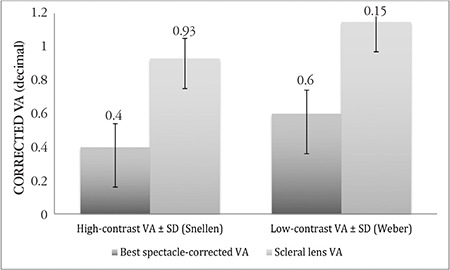
Evaluation of high- and low-contrast visual acuity after scleral lens application VA: Visual acuity, SD: Standard deviation

The study included 10 men and 14 women with a mean age of 25.2±5.9 (range: 17-36) years. Preoperative mean keratometry values were K1: 45.97±2.01 (range: 41.20-50.20) D, K2: 50.08±3.51 (range: 43.10-60.30) D, and Kmax: 57.51±5.18 (range: 48.60-69.80) D. Keratoconus was advanced in 72.4% of eyes (55.2% stage 3, 17.2% stage 4). High- and low-contrast VA improved significantly with the scleral lens (p<0.0001) (Figure 4). Mean central corneal clearance measured by OCT was 120.7±24.5 µm. There was no correlation between keratometry values and the sagittal depth of the scleral lens (Table 1). The mean number of trial lenses required for a successful fit was 2.2 (range: 1-8) lenses. After scleral contact lens application, the patients reported high scores for comfort (mean score: 4.69; range: 4-5) and vision (mean score: 4.62; range, 3-5). The patients’ mean VAS score for overall satisfaction was 88.1 (range, 70-100).

## Discussion

Gas-permeable rigid contact lenses have been used for many years for visual rehabilitation in keratoconus. However, in patients with advanced keratoconus, anterior corneal irregularity leads to centration problems and application difficulties. For this reason, scleral lenses can be used successfully for the visual rehabilitation and management of a variety of corneal disorders in which adequate response is not achieved with other treatments. The main indication for scleral lenses is optical correction of an irregular corneal surface, especially due to keratoconus or corneal transplantation.^5,14^ In previous studies, visual results of 20/40 or better were reported in 91% of keratoconus patients.^1,15,16^ In our study, the patients’ visual acuity was 0.9±0.1 with the scleral lens. Most (72.4%) of the patients were stage 3 or 4 keratoconus and there were no corneal scars. Therefore, high VA was obtained after scleral lens application.

Some fitting difficulties associated with scleral lenses may limit their use. Compared to other lenses, scleral lenses are larger in diameter, take longer to apply, and are costly. Our clinical experience showed that scleral lens fitting with the use of standard trial sets may be comparable or easier than fitting corneal or corneoscleral rigid lenses. We used an average of 2 trial lens to achieve a successful fit. Similar to our results, this number is between 2 and 3.2 in the literature.^1,17,18^

OCT imaging has improved modern scleral lens fitting by providing accurate measurements for trial lens selection and contact lens fit assessment and preventing ocular complications associated with lens application. High-resolution imaging of the anterior segment has also provided more information on corneal and scleral morphology and physiology. Measuring central corneal clearance with OCT has allowed us to objectively determine the amount of settling that occurs over time.^19^ In addition, the use of OCT during fitting has enabled the evaluation of peripheral edge alignment and objective measurement of the central corneal opening.^10^ In our study, anterior segment OCT was used both to measure central corneal clearance (120 µm) and to visualize edge alignment. Topography data obtained from keratoconus patients before contact lens fitting can guide lens selection. In our study, there was no correlation between measured topography values of the patients’ eyes and the sagittal height of the lens. Therefore, scleral lenses can be fitted successfully even in the absence of topographic data.

Scleral lenses are expected to be more comfortable than gas-permeable rigid contact lenses because they rest on the sclera and do not touch the cornea. In a study by Yan et al.^18^, 91% of patients reported comfortable 10-hour daytime lens wear. In another study evaluating patient satisfaction, 78.9% comfort, 78.2% visual quality, and 87.7% overall satisfaction were reported.^20^ In our study, patients also reported high scores for comfort (93.8%), visual acuity (92.4%), and overall satisfaction (88.1%).

## Conclusion

In conclusion, scleral lenses are an important option that offers optical rehabilitation and comfort for keratoconus patients. The use of OCT is a valuable adjunct to traditional contact lens fitting techniques. It is also an easy and fast way to evaluate lens fit with relation to the cornea, limbus, and sclera.

## Figures and Tables

**Table 1:**
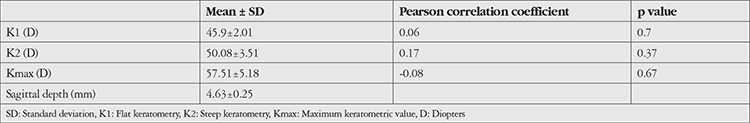
Correlation between sagittal height and keratometry values
